# A ten years (2000–2009) surveillance of resistant *Enterobacteriaceae* in Zhejiang Province, China

**DOI:** 10.3402/mehd.v23i0.11609

**Published:** 2012-03-23

**Authors:** Rong Zhang, Tomoaki Ichijo, Yan-Yan Hu, Hong-Wei Zhou, Nobuyasu Yamaguchi, Masao Nasu, Gong-Xiang Chen

**Affiliations:** 1 Second Affiliated Hospital of Zhejiang University, Zhejiang University, Hangzhou, China; 2 Graduate School of Pharmaceutical Sciences, Osaka University, Osaka, Japan

**Keywords:** long-term resistant surveillance, area distribution, carbapenems, Enterobacteriaceae

## Abstract

**Objective:**

In Zhejiang Province, there are several highly developed cities near the coast and several relatively under-developed mountain areas. Analysis of the composition of bacteria isolated from patients as well as their antibiotic resistance profile from various areas of this province, and tracing of such data year-by-year, will help to delineate the bacterial resistance profile of these areas and to understand how the stage of socio-economical development impacts on the composition of clinical micro-flora and their resistance profile.

**Methods:**

In order to investigate variation in resistance rates and isolation rates of *Enterobacteriaceae*, from 2000 to 2009 in Zhejiang Province, China, *Enterobacteriaceae* isolated from 15 hospitals located in different regions of the province were surveyed.

**Results:**

The total numbers of the *Enterobacteriaceae* isolated increased more than 20-fold from 2000 to 2009. Among the *Enterobacteriaceae*, *Escherichia coli* and *Klebsiella pneumoniae* were the dominant isolates. The percentage of *E. coli* and *K. pneumoniae* that produced detectable extended-spectrum β-lactamases (ESBLs) increased from 2000 to 2007, and then declined slightly in 2008 and 2009. The percentages of *K. pneumoniae* and *E. coli* that were resistant to ceftazidime increased sharply from 2000 to 2009. There were remarkable increases in the carbapenem resistant rates during the decade, but they were much higher for the isolates from the developed cities than from the rural areas. In 2002, carbapenem-resistant *E. coli* was first found in Hangzhou, one of the highly developed cities in Zhejiang Province. By 2009, carbapenem-resistant bacteria were found for all species of *Enterobacteriaceae* surveyed in almost all areas of the province, although they were more frequently identified in developed areas than in rural areas.

**Conclusion:**

Much restrictive actions have to be taken in terms of rational use of antibiotics and nosocomial control to prevent the further spread of the drug-resistant pathogens.

The reformation and opening up of China in the last two decades of the 20th century was accompanied by socio-economic development and a gradual improvement in the standard of living. The life style of the Chinese people, especially people living in the relatively developed areas of China, has undergone significant changes. Such changes have had a profound impact on micro-flora and resistant bacteria both in the environment as well as in clinical settings. One of the most important factors that contributed to these changes in micro-flora and to the resistance profile of bacteria was the abuse of antibiotics. Thus, antibiotics have been widely used and, in addition, over-doses of various antibiotics have been administered for healthcare and in farming where they are used as animal feed additives. The abuse of antibiotics in medical settings has directly impacted the resistance profile of clinically isolated bacteria. Moreover, antibiotics used for crop cultivation and/or in animal feeds not only alter micro-flora in the environment, which in turn impact on clinical micro-flora, but also increase the chance of exposure of humans to these antibiotics.

Zhejiang Province, which has a population of over 46 million, is located on the east coast of China and is the most economically developed province in China. While it is one of the largest areas in China for farming and aquaculture, this province also has the most highly developed medical system, with the highest medical consumption per capita among all of the provinces in China. However, the level of development among different areas even within this same province is not equal. There are several highly developed cities near the coast such as Hangzhou (the capital city of the province), Ningbo, Wenzhou, and Shaoxing, as well as several relatively under-developed mountain areas such as Lishui and Quzhou. Analysis of the composition of bacteria isolated from patients as well as their antibiotic resistance profile from various areas of this province, and tracing of such data year-by-year, will help to delineate the bacterial resistance profile of these areas and to understand how the stage of socio-economical development impacts on the composition of clinical micro-flora and their resistance profile.

Of the various bacteria that have been clinically isolated, gram-negative rods, especially the *Enterobacteriaceae*, are the most prevalent. The CHINET 2008 surveillance of bacterial resistance showed that *Enterobacteriaceae* account for 52% of clinical gram-negative bacteria, and that *Escherichia coli, Klebsiella pneumoniae, Enterobacter cloacae, Proteus mirabilis, Citrobacter freundii*, and *Serratia marcescens* were the most commonly isolated *Enterobacteriaceae* (1). *E. coli* accounts for 26.4–27.6% of gram-negative bacteria followed by *Klebsiella* spp. (13.8–19.6%) and *Enterobacter* spp. (4.7–5.8%) (1–4). Penicillin, cephalosporins, aminoglycosides, quinolones, β-lactam/β-lactamase inhibitor combinations, and carbapenems are currently used to treat *Enterobacteriaceae* infection. The *Enterobacteriaceae* that predominantly produce extended-spectrum β-lactamases (ESBLs) are *E. coli* and *K. pneumoniae*, and ESBL production was detected in close to 56.2 and 43.6%, respectively of these bacteria, consistent with previous studies (5, 6). ESBL production causes resistance to β-lactams and is usually mediated by plasmids. In addition to carrying β-lactamase genes, these plasmids may encode a number of aminoglycoside- or quinolone-modifying enzymes, which cause resistance to aminoglycosides and quinolones, respectively. Carbapenems that are stable in the presence of bacterially produced β-lactamases (including ESBLs and AmpC) showed strong activity against many gram-positive, gram-negative, and anaerobic bacteria (7, 8). Therefore, carbapenems are often used as the last choice for treatment of infections that are caused by multidrug-resistant *Enterobacteriaceae*.

In the present study, we focused on *Enterobacteriaceae* isolates from 15 hospitals located in widely different areas of Zhejiang Province. We conducted a retrospective investigation of the distribution of the isolates and their drug resistance profile, especially resistance to carbapenems, during the years 2000–2009 to gain an overview of the variation in bacterial drug resistance over this period.

## Materials and methods

### Hospitals that participated in the survey and bacterial isolates

Fifteen hospitals from 10 cities in Zhejiang Province participated in this survey: the Second Affiliated Hospital of Zhejiang University (Hangzhou), Zhejiang Provincial People's Hospital (Hangzhou), Zhejiang Provincial Hospital of Traditional Chinese Medicine (Hangzhou), Hangzhou First people's Hospital (Hangzhou), Hangzhou Third people's Hospital (Hangzhou), Zhuji People's Hospital of Zhejiang Province (Shaoxing); the First Hospital of Jiaxing (Jiaxing), Huzhou Central Hospital (Huzhou), Ningbo First Hospital (Ningbo), Quzhou Central Hospital (Quzhou), Dongyang People's Hospital (Jinhua), Lishui Central Hospital (Lishui), Taizhou Hospital of Zhejiang Province (Taizhou), Zhoushan Hospital (Zhoushan), the Second Affiliated Hospital of Wenzhou Medical College (Wenzhou). Isolates were collected from aseptically obtained body fluid such as blood, urine, pleural fluid, and ascites from both in-patients and out-patients during January 2000–December 2009.

### Antimicrobial susceptibility testing

The identity and susceptibility of isolates were confirmed using the VITEK system (bioMérieux, Hazelwood, MO, USA). *E. coli* ATCC25922 and *K. pneumoniae* ATCC700603 were used as reference strains for susceptibility testing. Meropenem and cefoperazone/sulbactam (2:1) were determined by the K-B method as recommended by the Clinical Laboratory Standards Institute (CLSI) of 2009 version (9). As no cefoperazone/sulbactam breakpoints are available for *Enterobacteriaceae*, susceptibility to cefoperazone was referred to in terms of resistance. All of the results were analyzed using WHONET 5.0 software (World Health Organization, Geneva, Switzerland).

### Phenotypic test for confirmation of ESBL-producing bacteria

ESBL-producing *Enterobacteriaceae* were confirmed using an ESBL confirmatory test according to CLSI guidelines (9). A 0.5 McFarland standard suspension of each isolate was inoculated on a Mueller-Hinton agar (MHA) plate as for the routine disk diffusion procedure. The plates were incubated for 16–18 h at 35°C. A ≥5 mm increase in a zone diameter for either antimicrobial agent tested in combination with clavulanic acid versus the zone diameter when tested in the absence of these agents was defined as ESBL-positive. *E. coli* ATCC25922 and *K. pneumoniae* ATCC700603 were used as reference strains for this ESBL confirmatory test.

### Polymerase chain reaction (PCR) and DNA sequencing

A total of 83 non-duplicated clinical carbapenem non-susceptible *Enterobacteriaceae* were collected from 10 areas of Zhejiang Province. The primers and reaction conditions used to amplify the *bla*KPC gene were as described previously (10). PCR products were sequenced using an ABI3730 Sequencer (Applied Biosystems, Foster City, CA), and the sequences were compared with the sequences reported in GenBank.

## Results

### Distribution of clinical isolates

Among the all bacteria isolated, *Staphylococcus aureus* was the most prevalent bacteria in the years 2000 and 2001. However, from 2002 and thereafter, it was replaced by *Pseudomonas aeruginosa* and followed by *Acinetobacter baumannii*. The most prominent *Enterobacteriaceae* bacteria in clinical isolates were *K. pneumoniae* and *E. coli* (Supplementary Table 1). Although there was no restrict identification of the origin of the isolates, it was estimated that more than 80% of the isolates were from in-patients, we suspect that majority of the isolates, especially with high resistance, were from the hospital-acquired infections.

**Supplementary Table 1:** Distribution of clinical isolates in Zhejiang Province from 2000 to 2009

No.	2000	No. of strains	2001	No. of strains	2002	No. of strains	2003	No. of strains	2004	No. of strains
1	*S. aureus*	773	*S. aureus*	1996	*P. aeruginosa*	2156	*P. aeruginosa*	2201	*P. aeruginosa*	4388
2	*P. aeruginosa*	767	*P. aeruginosa*	1782	*S. aureus*	1880	*S. aureus*	2025	*S. aureus*	2850
3	*E. coli*	501	*E. coli*	1084	*E. coli*	1190	*E. coli*	1695	*E. coli*	2594
4	*A. baumannii*	463	*A. baumannii*	984	*K. pneumoniae*	1069	*A. baumannii*	1585	*K. pneumoniae*	2466
5	*K. pneumoniae*	399	*K. pneumoniae*	941	*A. baumannii*	1035	*K. pneumoniae*	1387	*A. baumannii*	2465
6	*S. epidermidis*	336	*S. epidermidis*	770	*S. epidermidis*	811	*S. epidermidis*	1386	*S. epidermidis*	1922
7	*E. cloacae*	235	*E. faecalis*	456	*E. faecalis*	690	*E. faecium*	1123	*S. maltophilia*	1377
8	*E. faecalis*	228	*S. maltophilia*	428	*S. maltophilia*	621	*S. maltophilia*	792	*E. faecium*	1281
9	*S. maltophilia*	203	*E. faecium*	385	*F.meningosepticum*	500	*E. cloacae*	506	*E. faecalis*	769
10	*E. faecium*	108	*E. cloacae*	342	*E. cloacae*	483	*E. faecalis*	440	*E. cloacae*	768

No.	2005	No. of strains	2006	No. of strains	2007	No. of strains	2008	No. of strains	2009	No. of strains

1	*P. aeruginosa*	7225	*P. aeruginosa*	9502	*P. aeruginosa*	14667	*P. aeruginosa*	28049	*P. aeruginosa*	25897
2	*A. baumannii*	5875	*A. baumannii*	7819	*A. baumannii*	9577	*A. baumannii*	20892	*A. baumannii*	24938
3	*K. pneumoniae*	3438	*S. aureus*	4305	*K. pneumoniae*	6161	*S. aureus*	9234	*K. pneumoniae*	9472
4	*E. coli*	3003	*K. pneumoniae*	4058	*S. aureus*	5291	*K. pneumoniae*	8080	*S. aureus*	8033
5	*S. aureus*	2829	*E. coli*	2920	*E. coli*	3951	*E. coli*	6926	*E. coli*	6954
6	*S. epidermidis*	2394	*S. epidermidis*	2474	*S. epidermidis*	2880	*S. epidermidis*	4271	*S. maltophilia*	5155
7	*E. faecalis*	1262	*S. maltophilia*	1732	*S. maltophilia*	2344	*S. maltophilia*	4040	*S. epidermidis*	5036
8	*S. maltophilia*	1260	*E. faecium*	1386	*B. cepacia*	1942	*S. marcescens*	3809	*S. marcescens*	2997
9	*E. faecium*	1175	*B. cepacia*	1138	*E. faecium*	1674	*B. cepacia*	2193	*P. mirabilis*	2877
10	*B. cepacia*	1132	*E. faecalis*	990	*S. marcescens*	1339	*E faecalis*	1962	*E. faecium*	1798

### Distribution of clinically isolated *Enterobacteriaceae*

**Fig. 1:**
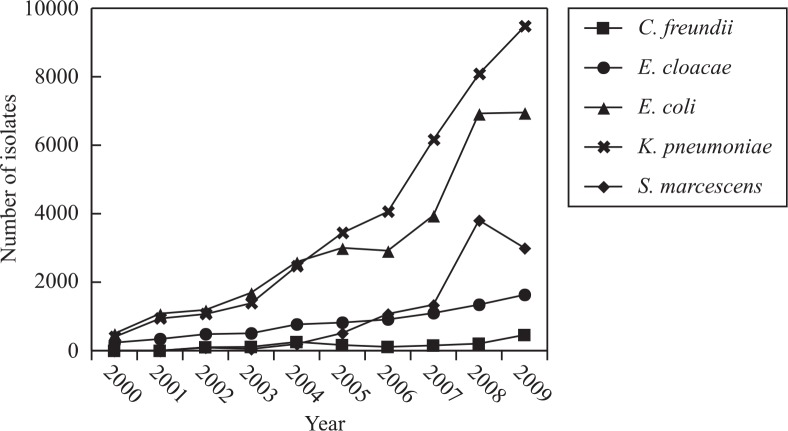
Distribution of clinically isolated *Enterobacteriaceae* in Zhejiang Province from 2000 to 2009.

The most commonly isolated *Enterobacteriaceae* (*E. coli*, *K. pneumoniae*, *E. cloacae*, *S. marcescens*, and *C. freundii*) are shown in Fig. 1. The number of isolated *Enterobacteriaceae* increased dramatically (about 20-fold) from 2000 to 2009. During the first 5 years of the survey (2000–2004), the number of *E. coli* was higher than that of *K. pneumonia*. From 2005 onward, *K. pneumoniae* was the most prevalent species of *Enterobacteriaceae*, followed by *S. marcescens* and *E. cloacae*.

### Detection of ESBL-producing bacteria

**Fig. 2:**
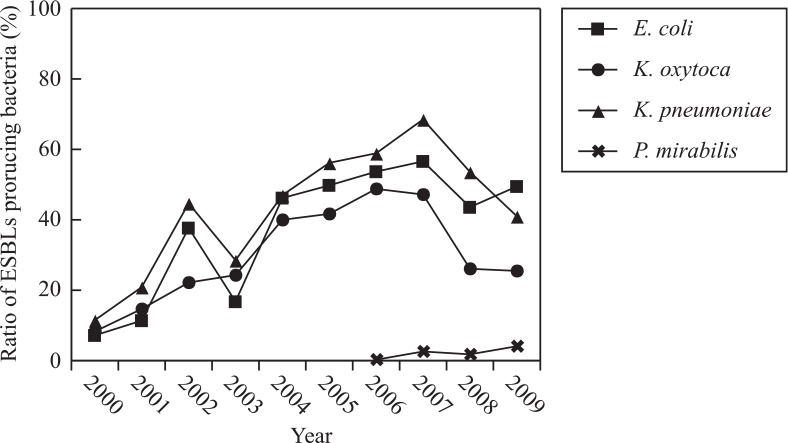
Annual changes in the detection ratio of *Enterobacteriaceae* that produced ESBLs from clinically isolated *Enterobacteriaceae*.

The main bacteria that produced ESBLs were *E. coli, K. pneumoniae, Klebsiella oxytoca*, and *P. mirabilis*. The percentage of *E. coli* and *K. pneumoniae* that produced ESBLs demonstrated a gradual and remarkable increase from 7.2% in 2000 to 56.6% in 2007, and from 11.5% in 2000 to 68.5% in 2007, respectively, while this percentage decreased in 2008 and 2009. ESBL-producing *P. mirabilis* was detected from 2006 onward (Fig. 2).

### Variation in the antimicrobial susceptibility of *Enterobacteriaceae*

**Fig. 3:**
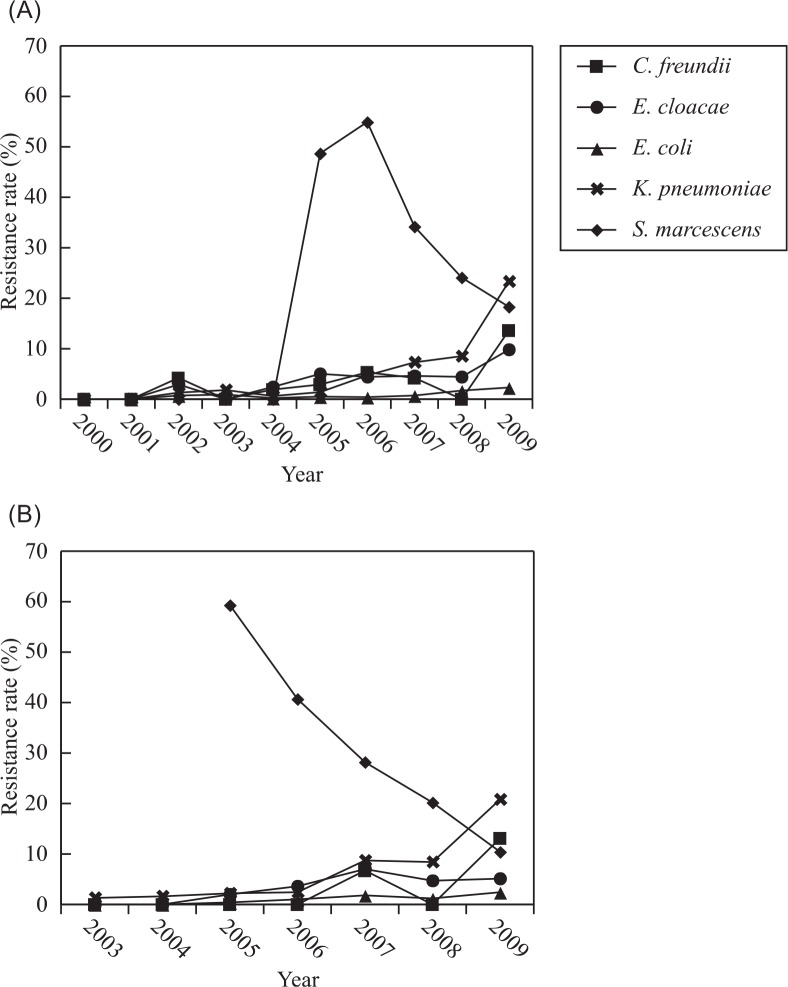
Annual changes in the resistance rate of various *Enterobacteriaceae* species against carbapenem from 2000 to 2009. (A) Imipenem, (B) meropenem.

The survey data (Supplementary Table 2) indicated that the percentage of bacteria that were resistant to ceftazidime increased sharply from 5.5% in 2000 to 45.3% in 2009. Some isolates were even resistant to carbapenems (imipenem and meropenem). Compared with *E. coli*, a higher percentage of *K. pneumoniae* showed resistance to antimicrobial agents. The percentage of resistant *K. pneumoniae* increased annually, in particular the percentage of carbapenem-resistant bacteria, which suddenly reached as high as 20.8% in 2009 (Fig. 3). While the percentage of *E. cloacae* that were resistant to cephalosporins (except for ceftizoxime) remained at about 50%, resistance rate to carbapenems increased yearly, but remained under 10%. Resistance rate of *S. marcescens* to carbapenem suddenly increased to 40–60% in 2005 and 2006 due to an outbreak of carbapenem-resistant *S. marcescens* as reported (11). Although the number of isolated *C. freundii* was lower than that of the aforementioned *Enterobacteriaceae* isolates, their resistance rates also increased annually, and they showed a similar resistance rate to cephalosporins as *E. cloacae*. The resistance rate of *C. freundii* to imipenem and meropenem also reached 13.6 and 13.1%, respectively, in 2009.

**Supplementary Table 2:** Variation of antimicrobial susceptibility of *Enterobacteriaceae* isolated from 2000 to 2009

	Resistance rate (%)									
	
Antimicrobial agents	2000	2001	2002	2003	2004	2005	2006	2007	2008	2009
*Escherichia coli*										
Total number of isolates	501	1084	1190	1695	2594	3003	2920	3951	6926	6954
Aztreonam	23.5	15.3	25.0	21.0	35.0	46.1	48.5	56.2	57.5	51.4
Cefepime	10.3	12.1	32.8	19.7	35.3	46.7	47.0	52.7	52.1	49.4
Cefoperazone-sulbactam	–	–	–	2.4	4.8	10.3	8.3	4.8	9.4	9.0
Cefotaxime	26.0	23.1	36.5	36.3	50.1	56.8	57.0	64.1	63.6	61.7
Cefoxitin	14.3	22.8	24.4	11.9	11.9	20.6	18.1	22.1	14.3	16.9
Ceftazidime	5.5	14.8	23.6	14.0	26.7	34.8	38.5	46.3	50.0	45.3
Imipenem	0	0	0.7	0.9	0.2	0.5	0.4	0.7	1.7	2.3
Meropenem	–	–	–	0	0	0.4	1.0	1.8	1.2	2.4
Piperacillin-tazobactam	–	–	–	7.7	12.6	10.5	10.0	15.0	8.7	12.6
*Klebsiella pneumoniae*										
Total number of isolates	399	941	1069	1387	2466	3438	4058	6161	8080	9472
Aztreonam	24.1	47.5	56.2	49.0	45.0	61.6	65.5	74.2	64.2	61.7
Cefepime	8.6	26.8	51.5	21.0	27.9	45.8	61.4	65.3	57.9	58.1
Cefoperazone-sulbactam	–	–	–	9.9	13.4	31.5	23.8	18.8	24.8	37.0
Cefotaxime	33.0	38.0	56.0	45.8	49.7	67.8	69.8	74.4	70.7	63.0
Cefoxitin	22.2	27.6	35.8	20.0	17.0	26.6	33.9	32.4	27.5	32.0
Ceftazidime	23.9	27.3	46.9	34.6	42.9	54.2	58.7	69.3	60.1	58.4
Imipenem	0	0	1.3	1.8	0.6	1.4	4.7	7.3	8.5	23.3
Meropenem	–	–	–	1.3	1.6	2.2	2.4	8.7	8.4	20.8
Piperacillin-tazobactam	–	–	–	28.1	40.8	43.7	47.0	46.1	38.6	49.0
*Enterobacter cloacae*										
Total number of isolates	235	342	483	506	768	803	943	1096	1358	1598
Aztreonam	62.5	58.6	53.2	60.3	55.2	66.1	57.6	51.6	50.7	48.8
Cefepime	24.5	27.5	44.2	29.8	32.6	41.4	31.3	33.2	27.1	25.1
Cefoperazone-sulbactam	–	–	–	6.1	12.4	25.3	18.5	9.4	18.7	13.6
Cefotaxime	57.4	65.0	65.5	43.5	57.9	66.9	51.9	53.9	49.3	46.8
Cefoxitin	–	92.7	95.9	89.3	83.9	89.4	95.5	94.8	96.0	94.5
Ceftazidime	47.9	53.9	51.7	56.0	53.7	58.0	48.8	44.9	48.5	49.5
Imipenem	0	0	2.9	0	2.4	5.0	4.4	4.6	4.4	9.8
Meropenem	–	–	–	0	0	2	3.6	7.0	4.7	5.1
Piperacillin-tazobactam	–	–		31.3	34.7	42.2	31.2	26.6	26.4	21.8
*Serratia marcescens*										
Total number of isolates	19	33	59	44	187	551	1057	1674	3809	2997
Aztreonam	–	–	–	–	2.3	50.9	52.5	66.9	48.1	53.7
Cefepime	–	–	–	–	–	25.4	30.6	31.9	26.7	48.0
Cefoperazone-sulbactam	0	0	0		0	0	26.1	11.3	21.5	16.7
Cefotaxime	0	0	0	0	0	44.3	45.9	60.0	48.2	49.6
Cefoxitin	–	10.0	50.0	28.6	43.5	61.9	60.2	60.1	67.8	62.9
Ceftazidime	0	0	0	0	0	4.2	15.6	7.9	12.0	26.4
Imipenem	–	0	0	0	0	48.7	54.9	34.2	24.1	18.3
Meropenem	–	–	–	–	–	59.3	40.7	28.2	20.2	10.4
Piperacillin-tazobactam	0	0	0		2.3	27.6	35.2	33.7	30.3	25.1
*Citrobacter freundii*										
Total number of isolates	29	52	90	118	245	142	93	136	231	447
Aztreonam	0	33.3	43.5	25.0	28.6	34.3	31.6	56.0	48.3	50.0
Cefepime	0	20.0	22.7	25.0	13.0	28.6	27.8	36.0	37.9	31.8
Cefoperazone-sulbactam	–	–	–	–	13.0	10.0	6.4	19.1	20.0	15.9
Cefotaxime	28.7	25.0	37.5	33.3	46.0	35.7	36.4	42.1	35.3	56.5
Cefoxitin	–	–	85.7	78.5	69.6	86.4	83.3	88.9	91.2	93.8
Ceftazidime	30.0	35.7	41.4	25.0	32.8	25.0	36.9	48.0	41.4	45.7
Imipenem	0	0	4.2	0	1.9	2.9	5.3	4.2	0	13.6
Meropenem	–	–	–	0	0	0	0	6.7	0	13.1
Piperacillin-tazobactam	–	–	–	–	21.8	24.3	31.6	40.0	20.7	39.1

### Distribution of carbapenem-resistant *Enterobacteriaceae*

**Fig. 4:**
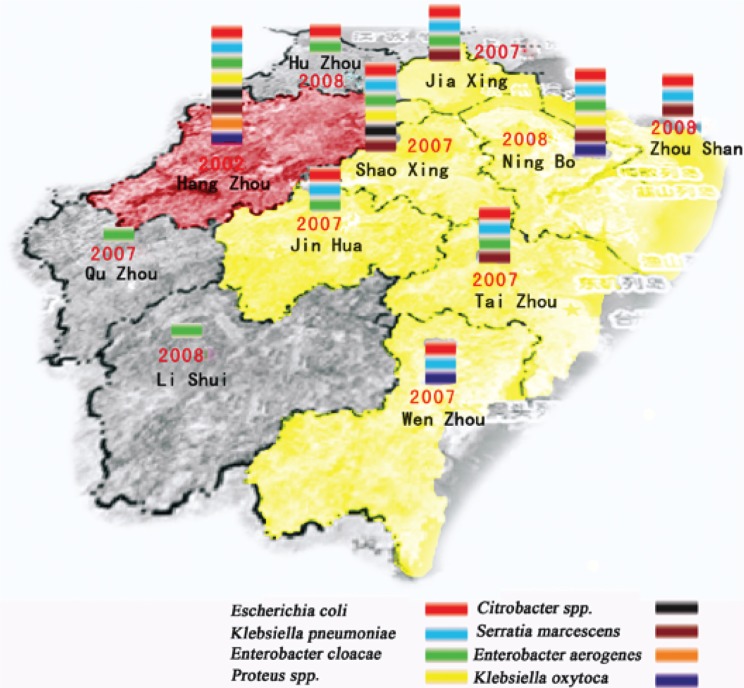
Prevalence of carbapenem-resistant *Enterobacteriaceae* from 10 areas in Zhejiang Province from 2000 to 2009. The year shown inside the area indicated the year when the first carbapenem-resistant *Enterobacteriaceae* was identified from this area. The sign of different color shown in the area indicates the different carbapenem-resistant *Enterobacteriaceae* so far have been identified from this area.

Carbapenem-resistant *Enterobacteriaceae* were identified from areas throughout the entire province (Fig. 4, Table 1). Since the first identification in 2002 of carbapenem-resistant *E. coli* in Hangzhou, the capital city of Zhejiang Province, eight species of carbapenem-resistant *Enterobacteriaceae* have been found in Hangzhou. It is clear from the map (Fig. 4) that there was a much higher number of carbapenem-resistant *Enterobacteriaceae* species in coastal areas than in inland areas. The most prevalent carbapenem-resistant *Enterobacteriaceae* were *E. coli, K. pneumoniae, S. Marcescens*, and *C. freundii*. The resistance rate to imipenem increased annually, especially in 2005–2007, when a high number of carbapenem-resistant *S. marcescens* was found in Hangzhou, while the resistance rate declined in 2008 and 2009. A previous study reported that all of the carbapenem-resistant *S. marcescens* isolated from Hangzhou belonged to the same clone (11).

**Table 1:** Detection of carbapenem-resistant *Enterobacteriaceae* in different districts of Zhejiang Province

District	Year of the earliest appeared carbapenem-resistant *Enterobacteriaceae*	Number of existing species of bacteria	Existing species of bacteria
Hangzhou district	In 2002, *E. coli*	8	*E. coli, K. pneumoniae, E. cloacae, P. mirabilis, C. freundii, S. marcescens, E. aerogenes, K. oxytoca*
Jiaxing district	In 2007, *K. pneumoniae*	4	*E. coli, K. pneumoniae, E. cloacae, S. marcescens*
Ningbo district	In 2008, *K. pneumoniae*	6	*E. coli, K. pneumoniae, K. oxytoca, E. cloacae, S. marcescens, P. mirabilis*
Shaoxing district	In 2007, *K. pneumoniae*	3	*E. coli, E. cloacae, K. pneumoniae, S. marcescens, C. freundii, P. mirabilis*
Huzhou district	In 2008, *E. coli*	2	*E. coli, E. cloacae*
Taizhou district	In 2007, *K. pneumoniae*	4	*E. coli, E. cloacae, K. pneumoniae, S. marcescens*
Lishui district	In 2008, *E. cloacae*	1	*E. cloacae*
Wenzhou district	In 2007, *K. pneumoniae*	3	*E. coli, K. pneumoniae, K. oxytoca*
Jinhua District	In 2007, *K. pneumoniae*	3	*E. coli, K. pneumoniae, E. cloacae*
Quzhou district	In 2007, *E. cloacae*	1	*E. cloacae*
Zhoushan district	In 2008, *K. pneumoniae*	3	*K. pneumoniae, E. coli, S. marcescens*

### Detection of blaKPC in Zhejiang Province

All of the 82 non-duplicated isolates including isolates of *E. coli*, *K. pneumoniae*, *E. cloacae*, *C. freundii*, *S. marcescens*, *Enterobacter aerogenes*, and *K. oxytoca* that were collected from different districts of Zhejiang Province harbored the *bla*KPC-2 gene, which can hydrolyze all β-lactams including carbapenems. This result indicates that *bla*KPC-2 is prevalent in the Zhejiang Province, which is thus a serious problem in terms of bacterial resistance.

### Variation in resistant bacteria among different geographic areas

**Fig. 5:**
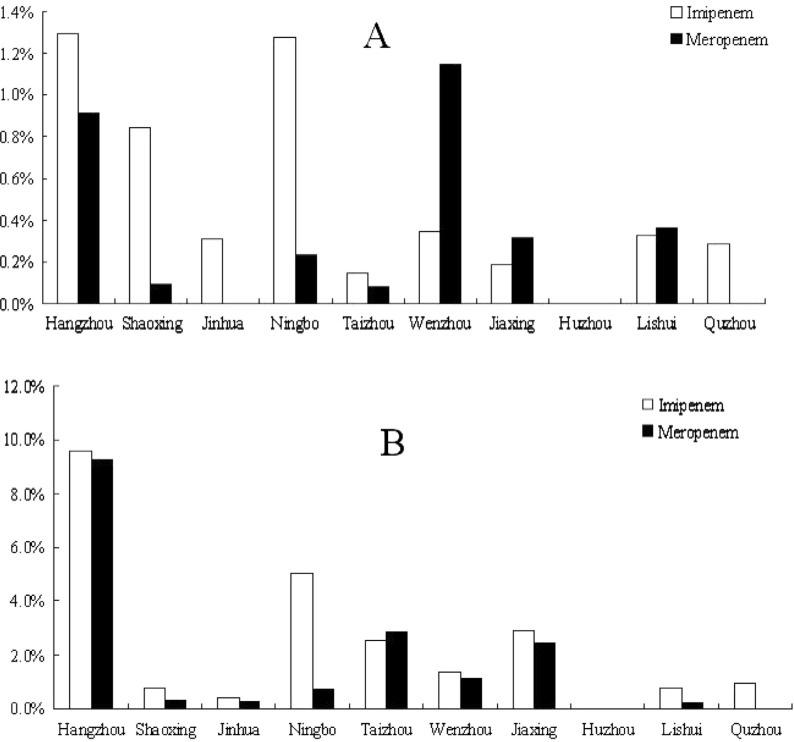
Resistant rates of *E. coli* (Panel A) and *K. pneumoniae* (Panel B) isolated from different areas of the province against imipenem (open bars) and meropenem (filled bars).

The distribution of this resistance differed according to geographic area. As shown in Fig. 5, resistance rates in economically developed areas (cities of Hangzhou, Ningbo, Shaoxing, Wenzhou, and Jinhua) were much higher than resistance rates in inland areas such as Lishui and Quzhou.

## Discussion

There was a remarkable increase in the total numbers of pathogens isolated over the last decade (Supplementary Table 1). There is about 15–20% ‘natural’ increase due to the increase in the number of patients. However, since 2007, there was a jump in the isolate numbers. This was attributed to the changes of the practice of blood-culture. Before that, blood culture was conducted for the blood of a single vial from one side. Since 2007, blood culture was required to be done for four vials (two vials on each side for both anaerobic and aerobic culture), which had remarkably increased the number of isolates. The emergence of ESBL-producing and carbapenem-resistant *Enterobacteriaceae* has made the treatment of infections caused by such bacteria a serious challenge. Carbapenem-resistant strains belonging to *E. coli* and *K. pneumonia* (12), the widely reported ‘super bacteria’, are also currently being clinically isolated. A survey of the resistance of *Enterobacteriaceae* in Zhejiang Province showed that *E. coli* and *K. pneumoniae* were the dominant *Enterobacteriaceae* isolated, and the number of clinical isolates belonging to these species has increased annually. From 2000 to 2004, *E. cloacae* were in the top 10 isolated species, whereas, after 2005, their numbers declined. In contrast, isolates of *S. marcescens* reached the top 10 starting in 2007, and these isolates were mainly isolated from five hospitals in Hangzhou. Pulsed-field gel electrophoresis (PFGE) typing showed that most of these *S. marcescens* isolates belonged to the same clone. The *K. pneumoniae* species that were isolated from the Second Affiliated Hospital of Zhejiang University were classified into five dominant clones (11). Despite the low level of *C. freundii* that was isolated, its drug resistance in both clinical and environmental settings is worthy of further attention.

The resistance survey also indicated that the most active aminoglycosidic agent against these isolates was amikacin, and the resistance rate to this agent remained below 30%. Resistance rate to amikacin even decreased for some species of *Enterobacteriaceae* (*E. coli, K. pneumoniae, E. Cloacae*, and *C. freundii*) over this decade. Resistance rate to gentamicin also declined over this decade. However, the percent of *E. coli* resistant to gentamicin remained at over 50%, which was higher than the resistance rate of *K. pneumoniae*. A high percentage of *E. coli* (60%) was also resistant to ciprofloxacin and levofloxacin, and this resistance rate was higher than that of other *Enterobacteriaceae* isolates, consistent with the 2008 CHINET survey of bacterial resistance in China (1).

The percentage of *E. coli* and *K. pneumoniae* that produced ESBLs gradually increased during this decade. The percentage of *E. coli* that produced ESBLs increased from 7.2% in 2000 to 56.6% in 2007, and then declined slightly. The percentage of *K. pneumoniae* that produced ESBLs remained higher than that of *E. coli*. These results explain why the resistance rate of *E. coli* to cephalosporin was lower than that of *K. pneumoniae*. The resistance rate of *E. coli* to ceftazidime and cefepime clearly increased over the decade, from 5.5 and 10.3% in 2000 to 50 and 52.1% in 2008, respectively. The resistance rate of *K. pneumoniae* to cefepime increased from 8.3% in 2000 to 65.3% in 2007, and then declined slightly. The resistance rate of *E. cloacae*, *S. marcescens*, and *C. freundii* to cephalosporin increased slightly over the survey period. According to the CLSI guidelines (9), new breakpoints against third-generation cephalosporins have been revised, which means that detection of ESBLs is now not required in routine clinical analysis. Further surveys should be carried out to monitor and evaluate the effectiveness of the new breakpoint guidelines.

The Study for Monitoring Antimicrobial Resistance Trends (SMART) in North America, Europe, Latin America, Middle East, Africa, and Asia from 2002 to 2007 showed that imipenem and meropenem showed good activity against *Enterobacteriaceae* (6). Similar results were also reported from other antibiotic resistance surveys. However the resistance rate to carbapenems also increased slightly during the decade (5, 13, 14). Our results were consistent with these previous studies. Carbapenem-resistant *K. pneumoniae* and *E. coli* were found in 2002, and carbapenem-non-susceptible *P. mirabilis* was subsequently found in 2004 (15). All β-lactams including carbapenems can be hydrolyzed by *bla*KPC. KPC was first detected in *K. pneumoniae* in 2001 (10) and was later found in other bacteria, such as other *Enterobacteriaceae* species, *P. aeruginosa, Pseudomonas putida, Acinetobacter* spp. and *Raoultella* spp. (16–18). *bla*KPC-2 was first reported in *K. pneumoniae* in 2007 in Zhejiang, China (19), although carbapenem-resistant *E. coli* was found in Hangzhou, in the Zhejiang Province, as early as 2002. However, insufficient attention was paid to this phenomenon. Since then, also other carbapenem-resistant *Enterobacteriaceae* such as *K. pneumoniae, E. cloacae, P. mirabilis, C. freundii, S. marcescens, E. Aerogenes*, and *K. oxytoca* have emerged in Zhejiang.

In addition, the percentage of carbapenem-resistant *K. pneumoniae* that was detected was higher than that of *E. coli*, especially in 2009. Moreover, PFGE typing showed that the *S. marcescens* isolates that were isolated in hospitals in Hangzhou during the epidemic of carbapenem-resistant *S. marcescens* that occurred in 2005–2007 belonged to the same clone, and most of them harbored *bla*KPC-2 (20). Resistance rate to carbapenems decreased remarkably in the following two years. This decrease was attributed to the following two factors. One was enforcement of taking various actions for prevention of bacterial infection such as separating the pathogen carriers and enforcement of hand sanitization of medical professionals by the government through the Nosocomial Infection Control Committee of the province. The second one was the restriction and control of the use of antibiotics by the Chinese Ministry of Hygiene, which has implemented guidelines for the rational use of antibiotics since 2006. In addition to resistance to carbapenems, the resistance rate of *Enterobacteriaceae* to antibiotics containing enzyme inhibitors (such as sulbactam or tazobactam) also increased over the survey period, and this trend toward increased resistance accorded with the increased resistance to carbapenems. For instance, both *K. pneumoniae* and *S. marcescens* showed high resistance to carbapenems, and their resistance rate to antibiotics containing enzyme inhibitors was also very high (2005–2007).

The geographic distribution of carbapenem-resistant *Enterobacteriaceae* indicated that more resistant isolates were identified from coastal and developed cities than from rural and mountain areas, and that the resistance rate of the isolates from developed cities was much higher than that from rural areas. Hangzhou (the capital city), Wenzhou, Ningbo, and Taizhou are coastal cities, while Shaoxing is an industrially developed area. These areas and cities are economically developed with higher income per capita and more frequent use of antibiotics, especially of broad-spectrum antibiotics, than rural areas such as in Lishui and Quzhou. Economically developed areas have a relatively developed medical system with a higher chance of antibiotic exposure that will increase the possibility of bacterial resistance. Moreover, the higher population density in these areas also increases the chance that resistant pathogens will be transferred among the population. While social-economic development has helped to improve the living standard of the people, it has also changed the micro-flora, especially the pathogens, and increased bacterial resistance, which poses a challenge to our future medical care and infection-control strategies. This finding is in line with our previous report that, during the last several decades, the ratio of *Shigella spp*. (*Shigella flexneri* vs. *Shigella sonnei*) in the province has undergone a shift from a ratio that is typical of developing countries to a ratio that is typical of industrialized countries (21).

Currently, the emergence of multi-resistant gram-negative bacteria, especially of *Enterobacteriaceae*, is a serious problem in Zhejiang, and should be further monitored in the coming years. Much restrictive actions have to be taken in terms of rational use of antibiotics and nosocomial control to prevent the further spread of the multi-resistant pathogens.
